# Inhibitory effect of green coffee bean extract on fat accumulation and body weight gain in mice

**DOI:** 10.1186/1472-6882-6-9

**Published:** 2006-03-17

**Authors:** Hiroshi Shimoda, Emi Seki, Michio Aitani

**Affiliations:** 1 Oryza Oil & Fat Chemical Co., Ltd., Research & Development Division, 1 Numata Kitagata-cho, Ichinomiya, Aichi 493-8001, Japan

## Abstract

**Background:**

An epidemiological study conducted in Italy indicated that coffee has the greatest antioxidant capacity among the commonly consumed beverages. Green coffee bean is rich in chlorogenic acid and its related compounds. The effect of green coffee bean extract (GCBE) on fat accumulation and body weight in mice was assessed with the objective of investigating the effect of GCBE on mild obesity.

**Methods:**

Male ddy mice were fed a standard diet containing GCBE and its principal constituents, namely, caffeine and chlorogenic acid, for 14 days. Further, hepatic triglyceride (TG) level was also investigated after consecutive administration (13 days) of GCBE and its constituents. To examine the effect of GCBE and its constituents on fat absorption, serum TG changes were evaluated in olive oil-loaded mice. In addition, to investigate the effect on hepatic TG metabolism, carnitine palmitoyltransferase (CPT) activity in mice was evaluated after consecutive ingestion (6 days) of GCBE and its constituents (caffeine, chlorogenic acid, neochlorogenic acid and feruloylquinic acid mixture).

**Results:**

It was found that 0.5% and 1% GCBE reduced visceral fat content and body weight. Caffeine and chlorogenic acid showed a tendency to reduce visceral fat and body weight. Oral administration of GCBE (100 and 200 mg/kg· day) for 13 days showed a tendency to reduce hepatic TG in mice. In the same model, chlorogenic acid (60 mg/kg· day) reduced hepatic TG level. In mice loaded with olive oil (5 mL/kg), GCBE (200 and 400 mg/kg) and caffeine (20 and 40 mg/kg) reduced serum TG level. GCBE (1%), neochlorogenic acid (0.028% and 0.055%) and feruloylquinic acid mixture (0.081%) significantly enhanced hepatic CPT activity in mice. However, neither caffeine nor chlorogenic acid alone was found to enhance CPT activity.

**Conclusion:**

These results suggest that GCBE is possibly effective against weight gain and fat accumulation by inhibition of fat absorption and activation of fat metabolism in the liver. Caffeine was found to be a suppressor of fat absorption, while chlorogenic acid was found to be partially involved in the suppressive effect of GCBE that resulted in the reduction of hepatic TG level. Phenolic compounds such as neochlorogenic acid and feruloylquinic acid mixture, except chlorogenic acid, can enhance hepatic CPT activity.

## Background

Coffee is one of the most commonly consumed beverages worldwide. However, the stimulating effect of caffeine on the central nervous system has significantly reduced the frequency of consumption due to its side effects on the cardiovascular system [1], central nervous system [2], and endocrine system [3]. On the other hand, scientific studies have revealed that both coffee and caffeine play a preventive role against various degenerative diseases of modern society. Van Dam and Feskens [4] reported that moderate daily consumption of coffee helped to reduce the risk of type 2 diabetes, while Fredholm and Lindgren found that caffeine promotes lipolysis in rat adipocytes [5]. Human studies show that caffeine enhances energy expenditure [6] and improves the clinical conditions of diabetic patients [7,8]. Another study by Greer *et al. *revealed that caffeine ingestion promotes glucose consumption with an increase in blood epinephrine [9], while pre-exercise consumption promotes ventilation and enhances lipolysis [10]. Chlorogenic acid, another main constituent of coffee beans, has recently been reported to selectively inhibit hepatic glucose-6-phosphatase [11], which is a rate-limiting enzyme involved in gluconeogenesis. However, roasting of coffee beans has been shown to reduce the content of chlorogenic acid in coffee [12]. Green coffee beans are rich in chlorogenic acid and its related compounds that show hypotensive effect [13]. In the present study, the effect of green coffee bean extract (GCBE) and its principal constituents on mice body weight and visceral fat contents were investigated. In addition, the effect of GCBE on fat absorption and metabolism were examined.

## Methods

### Animals

Male ddy mice (Japan SLC Inc., Shizuoka, Japan) were used for the experiments. The animals were housed at 23°C ± 1°C and were fed standard non-purified diet (CE-2, Clea Japan Inc., Shizuoka, Japan) and tap water *ad libitum*. The experiments were conducted in accordance with the Guidelines for Animal Experimentation (Japan Association for Laboratory Animal Science, 1987). All experimental designs were approved by the Ethical Committee for Use of Experimental Animals.

### Preparation and determination of GCBE

**Figure 1:**
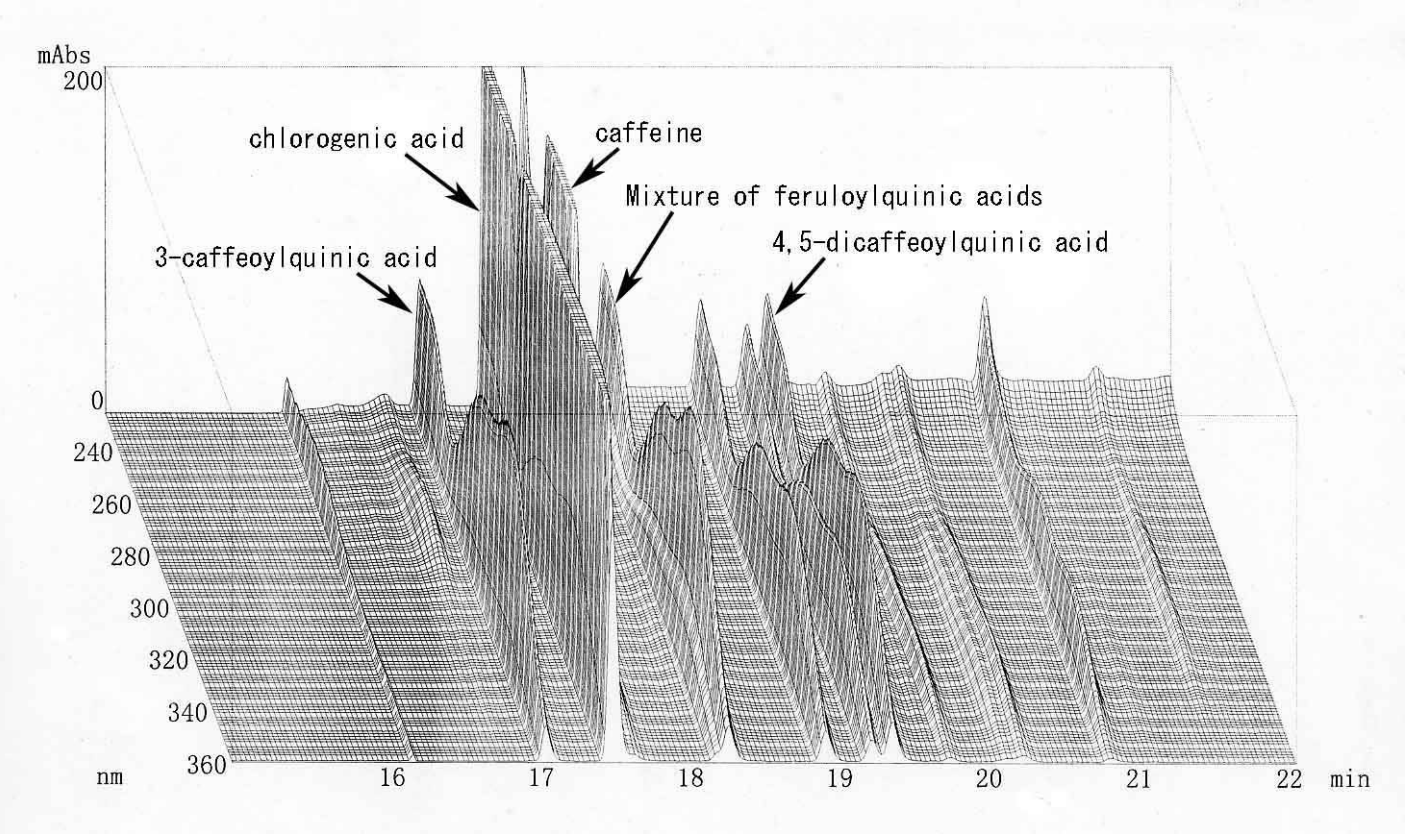
Three dimensional HPLC chromatogram of GCBE.

**Figure 2:**
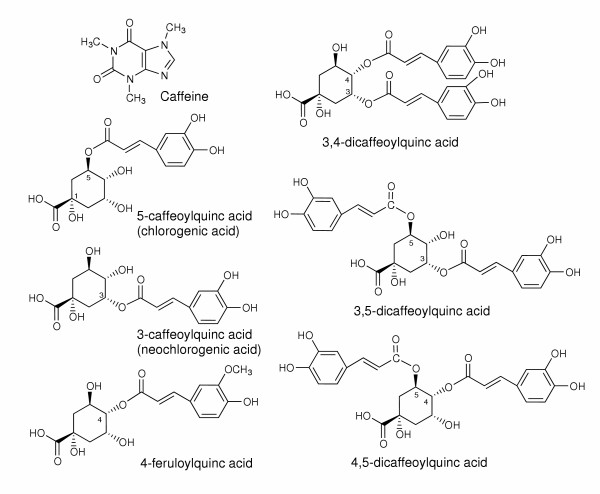
Chemical structures of constituents of GCBE.

GCBE (yield: 13.7%) was extracted from green coffee (*coffea canephora*) beans at 70°C for 2 h using 70% ethanol. Caffeine, chlorogenic acid and its related compounds were analysed by HPLC using anhydrous caffeine (Kishida Chemical Co., Ltd., Osaka, Japan) and chlorogenic acid (Sigma-Aldrich Co., Ltd., St. Louis, MO, USA) as standards (Figure 1). HPLC equipped with a Capcellpack C18 (4.6 φ × 250 mm, Shiseido, Tokyo, Japan) and a photodiode array detector (SPD-10 Avp, Shimadzu, Kyoto, Japan) was used. The solvent used included (A) 2 mM H_3_PO_4 _and (B) CH_3_CN; a linear gradient of (A) was changed to (B) after 35 min. The flow rate was maintained at 1.0 mL/min. The amounts of caffeine and chlorogenic acid detected were 10.0% and 27.0%. As shown in Figure 2, GCBE contains chlorogenic acid and other related compounds, namely, 3-caffeoylquinic acid (neochlorogenic acid) and a mixture of feruloylquinic acids. These compounds were isolated and identified from GCBE [14]. The amounts of 3-caffeoylquinic acid and a mixture of feruloylquinic acids using standard chlorogenic acid were 5.5% and 16.0%. The content of 4,5-dicaffeoylquinic acid was 5.2% using an authentic sample which was isolated from Japanese butterbur.

### Reagents

Sesamin (Industrial Research, Ltd., Wellington, New Zealand) and orlistat (Hoffman-La Roche Ltd., Basel, Switzerland) were used as reference compounds. EDTA was purchased from Kishida Chemical Co., Ltd. (Osaka, Japan), while palmitoyl CoA and L-carnitine were obtained from Sigma-Aldrich Co., Ltd. (MA, USA). Calcium and magnesium-free phosphate buffered saline [PBS (-)], Triglyceride E-test Wako, olive oil, sucrose, Triton X-100 and 5,5'-dithiobis(2-nitrobenzoate) (DTNB) were purchased from Wako Pure Chemicals Industries, Ltd., Osaka, Japan.

### Measurements of mice body weight and visceral fats

Six week-old mice were given free access to nonpurified diet (CE-2) containing 0.5% or 1% GCBE for 14 days. Caffeine (0.05% and 0.1%) and chlorogenic acid (0.15% and 0.3%) were added to the diet. Mice were weighed every 2 days. Mice epididymal and perirenal fats were removed and weighed at the end of the experiment. Orlistat, a medicine prescribed for obesity, was used as a positive control.

### Measurements of hepatic triglyceride level in mice

Acacia gum (5%) suspension containing GCBE (100 and 200 mg/kg· day), caffeine (10 and 20 mg/kg· day) and chlorogenic acid (30 and 60 mg/kg· day) was administered daily to 5 week-old mice for 13 days. On day 14, the livers were removed and approximately 200 mg of the liver was homogenized in 1 mL of PBS (-). This suspension was then centrifuged (2200 × g, 10 min), followed by the extraction of hepatic triglyceride (TG) using a mixture of chloroform and methanol (2:1). The extract was evaporated and resuspended in 1 mL PBS (-). The TG content was measured using Triglyceride E-test Wako. Sesamin was used as a positive control.

### Determination of serum TG in olive oil-loaded mice

The experiments were carried out as described by Shimoda *et al*. [15]. Initial blood samples were collected from infraorbital venous plexus of mice (aged 5 to 6 weeks) after fasting for 20–22 h. The mice were administered with an acacia gum (5%) suspension containing GCBE (200 and 400 mg/kg), caffeine (20 and 40 mg/kg) and chlorogenic acid (60 and 120 mg/kg). After 30 min, olive oil (5 mL/kg) was administered to the mice. Blood samples were collected at intervals of 2 h after loading with olive oil. The samples were centrifuged and serum TG was measured using Triglyceride E-test Wako. Orlistat was used as a positive control.

### Measurements of hepatic carnitine palmitoyltransferase activity in mice

Six week-old mice were fed a diet (CE-2) containing GCBE (0.5% and 1%), caffeine (0.05% and 0.1%), chlorogenic acid (0.15% and 0.3%), neochlorogenic acid (0.028% and 0.055%) and a mixture of feruloylquinic acids (0.081%) for 6 days. Mice livers were removed and homogenized in Tris-HCl buffer (pH 7.4) weighing 6- fold that of liver weight containing 0.25 M sucrose and 1 mM EDTA. The homogenate was centrifuged (880 × g, 4°C, 10 min) and the supernatant was collected; this was followed by a second centrifugation (11,770 × g, 4°C) to obtain mitochondrial fraction. The precipitate of the mitochondrial fraction was flushed and suspended in buffer to obtain a protein concentration of 6 mg/mL. CPT activity was measured according to the method described by Markwell *et al*. [16]. Tris buffer (58 mM, pH 8.0, 1 mL) containing 1.25 mM EDTA, 0.1% Triton X-100 and 0.25 mM DTNB, 37.5 μM palmitoyl CoA (20 μL) and 6 mg/mL mitochondrial fraction (20 μL) were mixed, and the optical density (OD) was recorded at 412 nm for 5 min. Subsequently, 1.25 mM L-carnitine (20 μL) was added, and the OD was recorded for 5 min. The CPT activity was calculated as follows:

CPT activity(nmol/min/mg protein)=(ΔODa−ΔODb)×20c×10,00013,000d×6e
 MathType@MTEF@5@5@+=feaafiart1ev1aaatCvAUfKttLearuWrP9MDH5MBPbIqV92AaeXatLxBI9gBaebbnrfifHhDYfgasaacH8akY=wiFfYdH8Gipec8Eeeu0xXdbba9frFj0=OqFfea0dXdd9vqai=hGuQ8kuc9pgc9s8qqaq=dirpe0xb9q8qiLsFr0=vr0=vr0dc8meaabaqaciaacaGaaeqabaqabeGadaaakeaacqqGdbWqcqqGqbaucqqGubavcqqGGaaicqqGHbqycqqGJbWycqqG0baDcqqGPbqAcqqG2bGDcqqGPbqAcqqG0baDcqqG5bqEcqGGOaakcqqGUbGBcqqGTbqBcqqGVbWBcqqGSbaBcqGGVaWlcqqGTbqBcqqGPbqAcqqGUbGBcqGGVaWlcqqGTbqBcqqGNbWzcqqGGaaicqqGWbaCcqqGYbGCcqqGVbWBcqqG0baDcqqGLbqzcqqGPbqAcqqGUbGBcqGGPaqkcqGH9aqpdaWcaaqaaiabcIcaOiabfs5aejabb+eapjabbseaenaaCaaaleqabaGaeeyyaegaaOGaeyOeI0IaeuiLdqKaee4ta8Kaeeiraq0aaWbaaSqabeaacqqGIbGyaaGccqGGPaqkcqGHxdaTcqaIYaGmcqaIWaamdaahaaWcbeqaaiabbogaJbaakiabgEna0kabigdaXiabicdaWiabcYcaSiabicdaWiabicdaWiabicdaWaqaaiabigdaXiabiodaZiabcYcaSiabicdaWiabicdaWiabicdaWmaaCaaaleqabaGaeeizaqgaaOGaey41aqRaeGOnayZaaWbaaSqabeaacqqGLbqzaaaaaaaa@7CE3@

ΔOD^a^: Linearity of optical density change for 1 min after addition of L-carnitine solution

ΔOD^b^: Linearity of optical density change for 1 min at baseline

c: Volume of mitochondrial fraction (μL)

d: Molar extinction coefficient of palmitoyl-CoA

e: Protein contents in mitochondrial fraction (mg protein/mL)

### Statistics

The results were expressed as mean ± SE. Significance of the differences was examined using the one-way ANOVA method, followed by Dunnett's test. Results with *p *< 0.05 were considered significant.

## Results

### Effect on body weight and visceral fat accumulation in mice

The changes in mice body weight and visceral fat content during the 14-day treatment with GCBE are tabulated in Table 1. The amount of diet intake was not reduced during the GCBE treatment. GCBE (0.5% and 1%) significantly suppressed mice body weight. The weight of mice epididymal and perirenal fats was significantly reduced in the group treated with GCBE (0.5%). However, the results for the effect of caffeine (0.05% and 0.1%) on the suppression of body weight and visceral fat were not significant. Chlorogenic acid (0.15% and 0.3%) demonstrated a weak suppressive effect on body weight and perirenal fat accumulation. Orlistat (0.1%), the positive control, showed a slight suppressive effect on epididymal and perirenal fat accumulation but no suppressive effect on body weight.

**Table 1:** Effects of GCBE and its constituents on body weight gain and accumulation of visceral fats

	Content in diet (%)	Mean total food intake (g/body)	Initial body weight Day 0 (g)	Final body weight Day 14 (g)
Control	--	75.3	28.2 ± 0.6	37.5 ± 1.6
GCBE	0.5	80.0	27.3 ± 0.5	33.2 ± 0.6 *
	1	75.5	26.9 ± 0.7	32.8 ± 1.2 **
Caffeine	0.05	74.9	27.0 ± 0.4	35.0 ± 1.0
	0.1	74.3	26.6 ± 0.4	33.9 ± 0.7
Chlorogenic acid	0.15	81.3	27.3 ± 0.5	35.2 ± 0.5
	0.3	76.5	26.4 ± 0.3	34.5 ± 0.5
Control	--	73.8	26.8 ± 0.4	34.6 ± 1.2
Orlistat	0.05	82.4	27.0 ± 0.2	35.0 ± 0.4
	0.1	81.3	26.3 ± 0.2	34.2 ± 1.0

	Content in diet (%)	Body weight gain (g)	Epididymal fat (mg)	Perirenal fat (mg)

Control	--	9.3 ± 0.4	605 ± 71	223 ± 39
GCBE	0.5	6.0 ± 0.1 *	393 ± 59*	106 ± 20 *
	1	5.8 ± 0.2 *	448 ± 68	124 ± 23
Caffeine	0.05	8.0 ± 0.2	536 ± 57	146 ± 29
	0.1	7.3 ± 0.2	491 ± 39	150 ± 20
Chlorogenic acid	0.15	7.9 ± 0.1	574 ± 48	173 ± 24
	0.3	8.1 ± 0.1	605 ± 82	175 ± 25
Control	--	7.8 ± 0.9	588 ± 55	249 ± 60
Orlistat	0.05	8.1 ± 0.4	612 ± 56	251 ± 45
	0.1	7.8 ± 0.9	481 ± 51 *	180 ± 33 *

Mice were fed non-purified diet (CE-2) containing each sample for 14 days. Each value represents the mean ± SE of 6 to 7 mice. The asterisks denote a significant differences from the control group, *:*p *< 0.05 and **: *p *< 0.01.

### Effect on hepatic TG accumulation in mice

**Figure 3:**
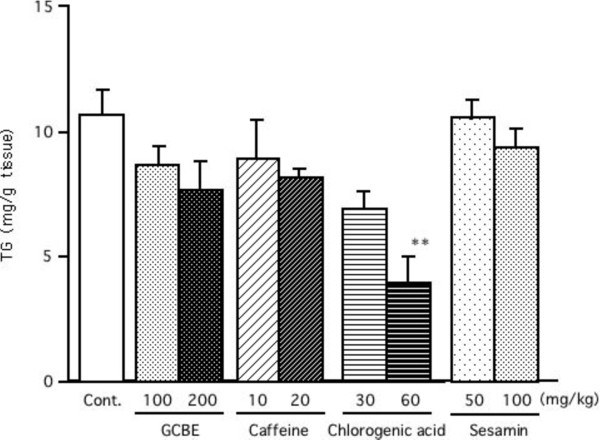
**Effects of GCBE, caffeine, chlorogenic acid and sesamin on hepatic TG accumulation in mice**. Each sample was administered once a day for 13 days. The columns represent the mean ± SE of 7 mice. The double asterisks denote a significant difference from the control group, **: *p *< 0.01.

The hepatic TG levels in mice were reduced after a 13-day treatment with GCBE, caffeine and chlorogenic acid. Figure 3 illustrates the effect of GCBE, caffeine and chlorogenic acid on hepatic TG. GCBE and caffeine showed a tendency to suppress hepatic TG. Further, the level of hepatic TG was significantly reduced by chlorogenic acid (60 mg/kg· day), suggesting its role in the suppression of hepatic TG in the group treated with GCBE.

### Effect on elevated serum TG level in olive oil-loaded mice

**Figure 4:**
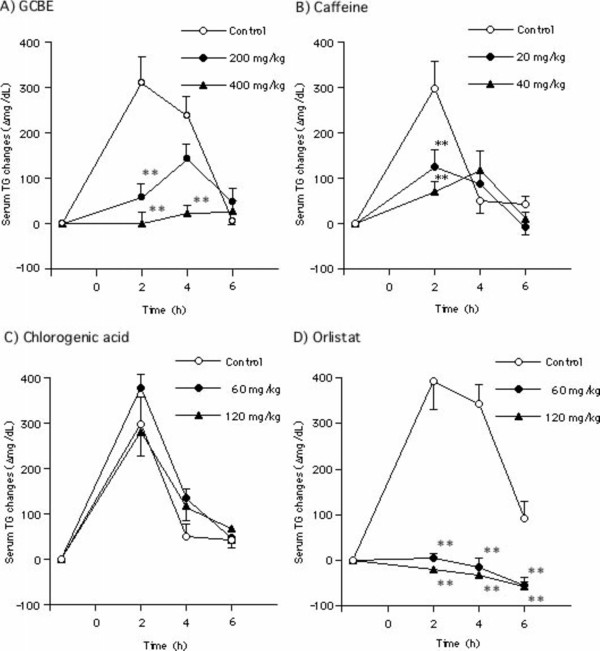
**Inhibitory effects of GCBE, caffeine, chlorogenic acid and orlistat on serum TG elevation in olive oil-loaded mice**. An initial blood sample was collected from each mouse, and each sample was orally administered 30 min later. After 1 h, olive oil was orally administered and a blood sample was collected every 2 h. Symbols represent the mean ± SE of 6 mice. The double asterisks denote a significant difference from the control group, **: *p *< 0.01.

The effects of GCBE and its constituents on fat absorption were examined in olive oil -loaded mice. The level of serum TG after fasting for 22 h was 60 to 125 mg/dL. The results showed that the elevated serum TG level was significantly lowered in groups treated with GCBE (200 and 400 mg/kg) as illustrated in Figure 4. Similarly, caffeine (20 and 40 mg/kg) also showed a suppressive effect on the increase in serum TG levels. However, chlorogenic acid at concentrations of 60 and 120 mg/kg did not affect serum TG levels. Caffeine is suggested to be the contributing compound for the suppression of fat absorption in GCBE-treated mice. Elevated serum TG level was completely suppressed by orlistat (60 mg/kg).

### Effect on hepatic CPT activity in mice

**Figure 5:**
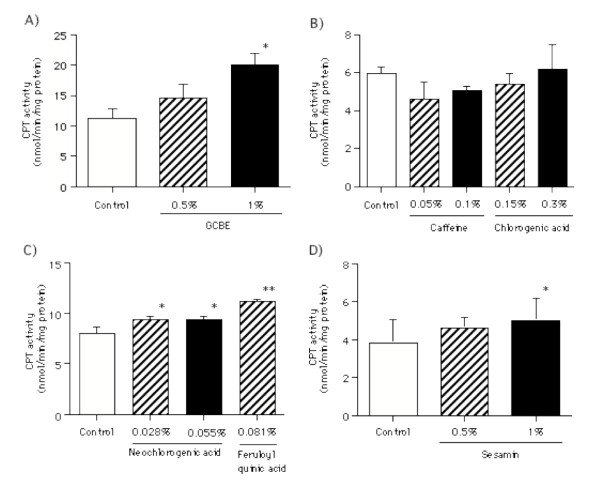
**Effects of GCBE, its constituents and sesamin on hepatic CPT activity in mice**. Mice were allowed free access to a diet containing each sample for 6 days. The CPT activity in the liver mitochondrial fraction was measured by the DTNB method. The columns represent the mean ± SE of 4–5 mice. The asterisks denote a significant difference from the control group, *: *p *< 0.05 and **: *p *< 0.01.

Part of the fatty acid metabolized and released from the adipose tissue is transferred to the liver for the oxidation process in the mitochondria of hepatocytes. CPT is a rate-limiting enzyme that catalyses the transportation of fatty acid to mitochondria for β-oxidation. As shown in Figure 5, GCBE (0.5% and 1%) demonstrated a dose-dependent enhancement of CPT activity in the liver mitochondria. Neochlorogenic acid (0.028% and 0.055%) and feruloylquinic acid mixture (0.081%) significantly enhanced CPT activity. However, caffeine and chlorogenic acid had no effect on CPT activity in the liver mitochondria. Sesamin (0.1%), the positive control, slightly enhanced the CPT activity.

## Discussion

The results showed that consumption of GCBE for 14 days caused a suppressive effect on weight gain and visceral fat accumulation in mice. GCBE contains 10% caffeine and 27% chlorogenic acid as the principal constituents, and these constituents showed a tendency to suppress body weight gain and visceral fat accumulation. Thus, these constituents are suggested to be partially involved in the suppressive effect of GCBE on body weight gain and visceral fat accumulation. Caffeine is known to be a lipolytic compound. On the other hand, the effect of chlorogenic acid on body weight gain has not yet been established. Rodriguez de Sotillo and Hadley reported that serum and hepatic TG levels were lowered with intravenous administration of chlorogenic acid in Zucker *fa/fa *rats [17]. However, the TG level in the adipose tissue was not lowered. Therefore, chlorogenic acid is suspected to be effective on hepatic TG, and not adipose TG. In our study, both pure caffeine and chlorogenic acid alone did not significantly suppress weight gain or visceral fat accumulation. Recently, it was demonstrated that catechin and caffeine in green tea showed synergistic effects on enhancing fat expenditure in obese mice [18]. Chlorogenic acid is also a dietary polyphenolic compound with antioxidative activity. Thus, it is suggested that caffeine, chlorogenic acid and other polyphenolic compounds in GCBE act synergistically to suppress body weight gain and visceral fat accumulation in mice.

Additional experiments were conducted to examine the effect of GCBE and chlorogenic acid on hepatic TG accumulation. Oral administration of chlorogenic acid (30 and 60 mg/kg· day) for 14 days reduced the level of hepatic TG in mice. However, the amount of visceral fat was unchanged (data not shown). The suppressive effect of chlorogenic acid on hepatic TG accumulation was more potent than that of GCBE. This result suggests that the TG-lowering effect of GCBE is partially due to chlorogenic acid. The other constituents of GCBE, such as cafestol and kahweol, which promote synthesis of lipids [19], may cause discrepancy in the activities of GCBE (crude chlorogenic acid) and pure chlorogenic acid.

Further studies were prompted to examine the anti-obesity effect of GCBE on dietary fat absorption using olive oil-loaded mice. The elevated serum TG level was lowered by GCBE and caffeine in olive oil-loaded mice. Coffee has been reported to delay gastric emptying [20]*via *constriction of smooth muscles in the proximal stomach. Hence, caffeine is suggested to delay fat absorption leading to lowering of elevated serum TG level.

Finally, the effect of GCBE and its constituents on hepatic CPT activity was examined. CPT activity was enhanced after 6-day consumption of GCBE. However, no effect was observed when similar examinations were conducted using caffeine and chlorogenic acid alone. Other polyphenolic compounds, namely, neochlorogenic acid and feruloylquinic acid, were isolated, and their effects on CPT activity were assessed. The chemical structures of neochlorogenic acid and feruloylquinic acid are quite similar to chlorogenic acid. The stereo configuration of carboxylic acid and caffeic acid or ferulic acid conjugated to the quinic acid moiety are suggested to be involved in the expression of the activity. Although the latest findings have revealed that sesamin [21] and γ-linoleic acid [22] enhance CPT gene expression, only a few reports have described the effects of dietary polyphenolic compounds. Moreover, their structure-activity relationship with regard to CPT activity has not been established. Further studies are required to determine the CPT enhancing activity of chlorogenic acid and its related compounds in GCBE.

## Conclusion

We conclude that GCBE can suppress body weight gain and visceral fat accumulation in mice. Caffeine suppresses fat absorption, while chlorogenic acid and its related compounds are found to be involved in the enhancement of fat metabolism in the liver.

## Competing interests

The author(s) declare that they have no competing interests.

## Authors' contributions

The experiments were designed by HS.

The experiments on animal models were performed by HS, ES and MA.

The determination of GCBE constituents was performed by HS and ES.

The manuscript was written by HS.

## Pre-publication history

The pre-publication history for this paper can be accessed here:


